# Sleep alterations just after extubation do not predict short-term respiratory failure, but…

**DOI:** 10.1186/s13613-021-00878-6

**Published:** 2021-05-26

**Authors:** Dominique Robert

**Affiliations:** Hospices Civils de Lyon, Médecine Intensive Réanimation, Lyon, France

In their recent paper, AW Thille et al. look for an association between sleep assessed by polysomnography (PSG) performed in the afternoon and the night after extubation and the occurrence or not of respiratory failure/reintubation. [1].

These authors, according to their previous works, have an actual interest and expertise both in weaning and sleep in ICU by PSG [2, 3]. In particular, in addition to usual sleep scoring they have identified an EEG pattern specific for ICU patients [4]. The rationale of this study is to find out if disturbed sleep during spontaneous ventilation just after extubation is associated with a risk of respiratory distress as it has been shown under mechanical ventilation before spontaneous ventilation trials [3, 5]. In the present work, including 52 patients considered at high risk of extubation failure, they also collected other recognized factors related to weaning outcome such as strengths of limb and respiratory muscles, respiratory drive, neurological functions (agitation scale and delirium screening). So the completeness of the data might allow a comprehensive analysis. Sleep is quantified by its duration and qualified as "normal" or as "atypical or no REM sleep". The results are that (a) sleep duration in the whole population recorded during 16 h [median] just after extubation is extremely reduced (2,4 h, median) and interestingly much more than before extubation under mechanical ventilation [3, 5] (b) there are no statistical differences as well in the weaning outcome in case of normal (*n* = 15) versus abnormal sleep (*n* = 37) as in sleep in case of respiratory failure (*n* = 12) versus no respiratory failure (*n* = 40). So, the initial hypothesis supporting that work is not confirmed. Beyond these results, some comments can be made:

1. Fundamentally that study is a physiological one aiming to positively rely sleep disturbances to respiratory performances and consequently to weaning outcome. But, the study was performed in a clinical setting, which obliges to offer the patients every validated treatment to guarantee the better outcome. Like that, NIV was applied, excepted during the PSG, in 50% of the patients raising a double risk. Firstly, to deprive of the NIV benefit during PSG recording (16 h); secondly, when applied to hide the potential role of sleep. That can be seen as an example of the difficulties in conducting physiological study and build its design in real life of clinical conditions.
2. Sleep visual analysis and scoring is a heavy task and have limitations as it appears in one study allowing comparison between visual and numeric analysis [5]. Particularly, automatic recognition of spindles, that allow to qualify sleep stage 2, is much more sensitive by using digital analysis. In the same way automatic analysis of the powers in different EEG frequencies allows to gradually distinguish different levels of wakefulness from full wakefulness to pathological one. So it is very likely that future study using such numeric in addition to visual analysis will allow better knowledge and understanding.
3. In the previous studies [3, 5] PSG were done during mechanical ventilation, whereas in the present study PSG were performed in spontaneous breathing straight after extubation. In that circumstance, the patient must face a drastically different task by providing the totality of the work of breathing. Specifically, such brutal necessity to adequately respond to the total respiratory load requires an as normal as possible response to PaCO2 and PaO2 and sufficient respiratory muscles functions. In normal subjects it is reported that sleep deprivation significantly decreases ventilatory control, respiratory motor output and endurance [6]. It is well probable that it is the same for ICU patients who are usually sleep deprived. It is also known that ICU-acquired diaphragmatic dysfunction is frequent and is positively related to weaning failure [7, 8]. So it could be appropriate change of view to consider that it is not the sleep which influences the weaning, but the weaning which changes the sleep. In case of seriously compromised ability to sustain wake spontaneous breathing sleep becomes a supplemental handicap by weakening sensibility to CO2 and decreasing respiratory muscles actions mainly during REM and deep sleep. So the vital priority may become to maintain respiration at the expense of sleep. It is of interest to notice that the short duration of sleep reported in the present study (2,4 h, median) in comparison to those reported under mechanical ventilation (range between 4 and 8 h) [3, 5] goes in that way. Even more, in the present study the shorter duration of sleep in case of no post extubation respiratory failure (2 h, median) than in case of respiratory failure (3,2 h, median), even if not statistically different, could be explained by the possibility to better resist to sleep pressure and stay awake in order to avoid respiratory failure.

## Data Availability

Not applicable.

## References

[1] Thille AW, Barrau S, Beuvon C (2021). Role of sleep on respiratory failure after extubation in the ICU. Ann Intensive Care.

[2] Thille AW, Richard JCM, Brochard L (2013). The decision to extubate in the intensive care unit. Am J Respir Crit Care Med.

[3] Thille AW, Reynaud F, Marie D, Barrau S, Rousseau L, Rault C (2018). Impact of sleep alterations on weaning duration in mechanically ventilated patients: a prospective study. Eur Respir J.

[4] Drouot X, Roche-Campo F, Thille AW, Cabello B, Galia F, Margarit L (2012). A new classification for sleep analysis in critically ill patients. Sleep Med.

[5] Dres M, Younes M, Rittayamai N, Kendzerska T, Telias I, Grieco DL (2019). Sleep and pathological wakefulness at the time of liberation from mechanical ventilation (SLEEWE). A Prospective Multicenter Physiological Study. Am J Respir Crit Care Med.

[6] Rault C, Sangare A, Diaz V, Ragot S, Frat JP, Raux M (2020). Impact of sleep deprivation on respiratory motor output and endurance. A Physiological Study. Am J Respir Crit Care Med.

[7] Levine S, Nguyen T, Taylor N, Friscia ME, Budak MT, Rothenberg P (2008). Rapid disuse atrophy of diaphragm fibers in mechanically ventilated humans. N Engl J Med.

[8] Qian Z, Yang M, Li L, Chen Y (2018). Ultrasound assessment of diaphragmatic dysfunction as a predictor of weaning outcome from mechanical ventilation: a systematic review and meta-analysis. BMJ Open.

